# The Fetal Origins of the Metabolic Syndrome: Can We Intervene?

**DOI:** 10.1155/2012/482690

**Published:** 2012-09-17

**Authors:** Noelle Ma, Daniel B. Hardy

**Affiliations:** ^1^The Department of Physiology and Pharmacology, The University of Western Ontario, London, ON, Canada N6A 5C1; ^2^The Department of Obstetrics & Gynecology, The University of Western Ontario, London, ON, Canada N6A 5C1; ^3^The Children's Health Research Institute, The Lawson Health Research Institute, London, ON, Canada N6A 4V2

## Abstract

Epidemiological studies have suggested that metabolic programming begins during fetal life and adverse events *in utero* are a critical factor in the etiology of chronic diseases and overall health. While the underlying molecular mechanisms linking impaired fetal development to these adult diseases are being elucidated, little is known about how we can intervene early in life to diminish the incidence and severity of these long-term diseases. This paper highlights the latest clinical and pharmaceutical studies addressing how dietary intervention in fetal and neonatal life may be able to prevent aspects of the metabolic syndrome associated with IUGR pregnancies.

## 1. Introduction

Clinical studies in humans have demonstrated that an adverse *in utero* environment (i.e., placental insufficiency-intrauterine growth restriction (IUGR)) contributes to long-term programming events leading to the metabolic syndrome, and ultimately, cardiovascular disease (CVD) [1–3]. This is of great interest considering that the incidence of IUGR (defined as birth weight below the 10th percentile) worldwide is estimated to be 15.5%, and that number is greatly underestimated [4]. Moreover, the incidences of noncommunicable diseases such as heart disease, type II diabetes, hypertension, obesity are on the rise in North America [5–7], with more than one in three Americans exhibiting obesity [8]. Although the prevalence of these chronic and noncommunicable diseases puts tremendous strain on the health care system and society, intervention with diet or drugs may play a significant role to reduce their incidence. For example, a meta-analysis study, using data from 58 clinical trials as well as nine cohort studies, indicates that in patients with vascular disease, a 1.8 mM reduction in LDL cholesterol by statins resulted in a 17% reduction in stroke and a 60% reduction in the risk of ischemic heart disease [9]. 

The problem is that current treatment for these diseases relies on the long-term use of pharmaceuticals in adults, which are not always efficacious for all individuals. For example, therapies using the statin class of lipid-lowering drugs to reduce hepatic cholesterol production have been successful in lowering LDL cholesterol by 24–61% [10]. However, while statin therapies are considered safe and effective in high doses, statins can lead to side effects including rhabdomyolysis, renal dysfunction, diabetes, and elevated liver enzymes [11]. This implies the need for additional strategies in disease prevention, not treatment.

Experiments of intrauterine growth restriction (IUGR) in animal models provide further evidence to support the hypothesis that impaired growth *in utero* via various maternal deficiencies leads to impairment of glucose, cholesterol, and triglyceride metabolism in adulthood [12–15]. *In utero* deficiencies that can lead to impaired growth in humans and animals include hypoxia [16], deficiencies in essential vitamins and minerals [17], diminished protein [15], caloric restriction [18], and excess glucocorticoids [19, 20]. Although the correlation between impaired fetal growth and the risk for developing chronic disease in adulthood is undoubtedly strong, emerging human and animal studies are now investigating how we might be able to intervene in early life to reduce or prevent these long-term programming events. This paper aims to look at the current literature to highlight the possible pharmaceutical and dietary intervention strategies to reduce the incidence of the metabolic syndrome long-term in patients from complicated pregnancies (i.e. low birthweight). 

## 2. Ascorbic Acid (Vitamin C) 

The maintenance of adequate antioxidant systems in cells and tissues is essential to the defense system against free radicals and reactive oxygen species (ROS) [21]. When free radical generation overcomes the protective systems of the cell, it can lead to changes in DNA structure, enzyme activity, and distortion of cell structures [21, 22]. Vitamins are a nonenzymatic and modifiable component of a cell's defense system. Vitamin C, a water-soluble vitamin, directly protects against aqueous peroxyl radicals, inhibiting initiated lipid peroxidation, and scavenges free radicals [23–25]. Vitamin E, a lipid-soluble vitamin, is able to prevent lipid peroxidation and can act as an inhibitor of free radical chain reactions [26]. Moreover, vitamins C and E have been shown to act synergistically, as vitamin C is able to help to regenerate and maintain levels of vitamin E [27]. 

Vitamins C and E have been investigated for use as an intervention method with the goal of preventing adverse pregnancy outcomes. Poor maternal environments including malnutrition and preeclampsia which have both been linked to IUGR, all have characteristically been shown to increase oxidative stress [28]. IUGR offspring have also been found to exhibit significantly lower expression of antioxidants [29]. Interestingly, in a prospective cohort study, after adjusting for factors such as vitamin supplementation, vitamins C and E have been positively correlated with birth weight and length [30]. Although, a direct causation between increased oxidative stress and adverse pregnancy outcomes has not been fully established, improving the defense systems of cells and tissues appears to be a logical first step in pregnancy intervention [29]. 

 In a rodent model of diabetes-induced growth restriction, supplementations of vitamins C and E during pregnancy led to a decrease in markers of oxidative stress in offspring, but did not equally prevent fetal growth restriction [31]. Interestingly, in a rodent model of lipopolysaccharide (LPS) mediated IUGR, pre- or post LPS injection with vitamin C administration alleviated IUGR and attenuated lipid peroxidation. Pre-LPS treatment with vitamin C had a stronger effect by decreasing fetal death as well [32]. However, researchers noted that the timing of vitamin C administration was seemingly important, as vitamin C administration post-LPS injection decreased the effectiveness of a pre-LPS injection with vitamin C [32]. Vitamin C intervention appears to produce more promising outcomes when given prior to LPS-induced IUGR in rodent pregnancies. 

Similarly, vitamin C has been pursued in human pregnancy trials. The majority of studies focused on a subpopulation of women at risk of preeclampsia; a maternal inflammatory response is believed to be mediated by a ROS imbalance [33, 34]. It should be noted that IUGR is often a severe consequence associated with preeclampsia [35]. However, when vitamins C and E interventions were given to pregnant women, the results did not support the use of vitamins as a viable intervention in pregnancy. Researchers did not consistently find a difference in the risk of preeclampsia and did not observe a change in birth weight or risk of IUGR [33, 34, 36–38]. Interestingly, a decrease for the risk of developing preeclampsia and markers of oxidative stress was restricted to a population of high-risk pregnancies [39]. 

Finally, the safety of vitamin supplements during pregnancy remains questionable as two separate studies suggest intervention with vitamins leads to an increase in LBW and preterm births [33, 38]. Thus, it still remains premature to determine the universal effectiveness of vitamin C or E supplementation during pregnancy for all populations of women. 

## 3. Folic Acid 

 Folate, and its synthetic form, folic acid, acts as necessary cofactors for biochemical reactions, namely, the formation of S-adenosylmethionine, the main methyl donor for methylation. Folate plays an important role in cell growth and replication, as folate deficiency has been associated with inhibited cell growth, DNA repair, and the potentiation of oxidative stress leading to chromosomal abnormalities [40, 41]. The importance of folic acid during pregnancy was first discovered when it was found to substantially reduce the risk of developing neural tube defects if 400 *μ*g of folic acid was taken daily during the periconceptional period [42, 43]. 

 Given its key role as a methyl donor, intervention with folic acid may initiate a possible epigenetic mechanism of early programming. In a rodent model of maternal protein restriction, hypomethylation of hepatic genes was observed, followed by a subsequent increase in gene expression levels. Folic acid supplemented to the restricted diet was able to prevent these epigenetic changes from occurring. It is conceivable that folic acid supplementation increases the availability of methyl groups for methylation [44]. Although providing additional methyl donors appears to prevent aberrant epigenetic changes, it is still important to determine whether additional methylation consistently translates into beneficial outcomes. For instance, Steegers-Theunissen et al. found that periconceptional intake of folic acid in mothers was directly related to an increase in methylation of the insulin growth-like factor 2 differentially methylated region (Igf2DM), which led to phenotypic consequences such as low birth weight [45]. 

 Since folic acid during pregnancy has been incorporated into Western diet, research has expanded to determine whether there are additional benefits conferred to pregnant women and their offspring [46]. Using a retrospective database, one study observed that women exposed to folic acid antagonists were found to be at a greater risk for severe preeclampsia, fetal growth restriction, and even death [47]. Thus, the availability of folic acid during pregnancy appears to be critical in obtaining positive pregnancy outcomes. For example, an observational study of 832 women highlighted that folate intake of less than or equal to 240 *μ*g from diet and/or supplements during pregnancy, doubled the risk of bearing a child with LBW [48]. This relationship remained significant even after controlling for confounding variables such as low-energy intake and maternal age [48]. Furthermore, in a subpopulation of women from Crete, Greece, it was found that daily intake of 500 *μ*g/day of folic acid during early to midgestation presented another window of opportunity to lower the risk for preterm delivery, LBW (<2500 g) and IUGR births, all risk factors for early programming of adult onset diseases [49]. Interestingly, when folic acid supplementation was consumed before conception, it also decreased the risk of LBW and IUGR births [50]. 

In light of these arguments, it is still important to look at the long-term health outcomes of offspring exposed to maternal folic acid supplementation. The Pune Maternal Nutrition Study, a community-based prospective study, investigated a group of 1102 pregnant rural Indian women, of which the majority exhibited low vitamin B12 levels [51]. Yajnik et al. found that in offspring at six years of age from mothers who had low vitamin B12 levels during pregnancy and were concomitantly exposed to high levels of folate had children that were more insulin resistant [51]. The authors studied other imbalances and found that children born to mothers with higher folate levels were associated with greater adiposity and insulin resistance, while low maternal vitamin B12 levels during pregnancy were attributed to children who became insulin-resistant long-term. Their study elegantly highlight a surprising and possibly harmful role of folic acid intervention during gestation and the early programming of type 2 diabetes. 

 To date, controlled studies involving folic acid intervention have produced variable results. For example, a double-blind trial by Fletcher et al. demonstrated that there was no difference observed in birth weight, placental weight, or gestational duration between a folic acid and iron supplementation compared to an iron supplementation alone in a population of English women [52]. Similar findings demonstrating a lack of association between folic acid and LBW have also been established in a nonanemic subpopulation of pregnant women [53]. Therefore, care must be taken when drawing conclusions from controlled studies investigating links between folic acid supplementation and pregnancy outcomes because many studies involve different interventions, subpopulations, and methodology. Baumslag et al. most vividly demonstrated this phenomenon through an early study, where folic acid intervention was administered to two different subpopulations, consisting of Bantu and Caucasian pregnant women in South Africa [54]. The Bantu women exhibited a decrease of almost four times the risk of delivering a child less than 5 lbs upon administration of 200 mg of iron and 5 mg of folic acid during pregnancy compared to an iron intervention alone. Interestingly, there were no benefits conferred to the Caucasian subpopulation on an average Western diet. The authors further suggest that folic acid supplementation would be most beneficial to target subpopulations with suboptimal diets [54]. 

 Although much research must still be pursued before folic acid intervention is used in complicated pregnancies, understanding the molecular mechanisms of folic acid actions will help to characterize its promising and beneficial effects. Given the key roles that folic acid play in cell growth, it has been hypothesized to play a role in early programming of long-term modifications. Hypomethylation of genes involved in cardiovascular and metabolic control in the liver following weaning was observed in a rat model of maternal protein restriction. However, supplementation with folic acid prevented hypomethylation and subsequent expression of these genes. It is conceivable that folic acid supplementation did increase the availability of methyl groups available for methylation [44]. 

 Having said that, it should be put into perspective that just because supplementation of folate in animal studies can improve a particular health-related outcome, it should not be concluded that supplementing folate will have an impact in the global prevalence of the problem. In addition, the toxicity of folic acid supplementation must also be considered before encouraging widespread use during pregnancy. High folate levels have been associated with decreases in nonspecific immunity and cancer promotion [55, 56].

## 4. Multiple Micronutrients

Maternal health and nutritional status have been considered one of the largest categories linked to perinatal morbidity [57]. Specifically, the level of micronutrients in the maternal diet can affect several pregnancy outcomes such as birth weight, gestational age at delivery, and perinatal mortality [58]. Often depending on the region, micronutrient deficiency may stem from an inadequate intake of animal source foods, an avoidance of milk or the influence of genetic polymorphisms that impair absorption or metabolism of nutrients [59]. Individual nutrient deficiencies have been explored including anemia development stemming from a lack of iron [60]. In addition, zinc deficiency is associated with preterm delivery and congenital abnormalities [60, 61]. 

 There are several approaches that can be undertaken to improve maternal nutritional status, including an increase in foods that are high in micronutrient content or nutrient supplements, the latter of which are mainly employed in research [59]. However, micronutrient deficiencies often coexist, particularly in developing countries [62]. This feature has led to the development of multiple micronutrient (MMN) supplements in hopes of providing multiple benefits through a single intervention [62]. UNICEF, United Nations University, and the World Health Organization have produced supplements containing 15 micronutrients present at doses that are sufficient to meet the needs of pregnant women in developing countries [63]. 

Intervention studies using MMN supplementations have been pursued largely in developing countries and have produced mixed results. In Tanzania, 1075 HIV-1-infected pregnant women received daily MMN supplementation without vitamin A or vitamin A supplementation alone during gestation [64]. The multivitamin supplementation was able to decrease the risk of preterm births, LBW and IUGR at birth, while vitamin A alone did not affect these outcomes. Women who consumed multivitamins gave birth to heavier babies compared to those receiving vitamin A alone [64]. In Nepal, daily MMN supplementation led to an increase in body weight of offspring when compared to folic acid and iron supplementation alone. Multivitamin supplementation was associated with higher birth weight of offspring while gestational duration was unaffected [65]. This study targeted a mix of both rural and urban individuals likely representing a more common subgroup of the population. In a follow-up study in Nepal, children who were exposed to MMN prenatally were evaluated two to three years later. The weight gain observed at birth persisted into childhood [66]. In contrast, in a study carried out in Mexico, among a subgroup of relatively healthy women exposed to nearly daily MMN supplementation, the birth size of offspring was no larger compared to iron alone [67]. Although these results directly contradict previous studies, it is important to note that the formula of MMN supplementations differed slightly making direct comparisons complex. For example, in the Tanzanian study, the supplements included zinc, which was not present in the Mexican study. 

In contrast to previous studies, Mathews et al. undertook a large-scale observational study on a population of pregnant women from an industrialized country, England [68]. Researchers observed no clinical effect of maternal nutrition on placental or birth weight at term. Moreover, vitamin C was the only nutrient that was found to have a positive correlation with placental and birth weight. However, researchers were skeptical that placental weight gain was clinically relevant. Together, these studies highlight the difference in efficacy of MMN supplementations and bring to light the importance of the population of women being studied. 

Interestingly, researchers have begun focus on micronutrient rich foods and pregnancy outcomes. In a prospective study of 797 rural Indian women, Rao et al. demonstrated that birth size was related to intake of green leafy vegetables at 28 weeks of gestation and milk consumption at 18 weeks of gestation [69]. In the same way, a study on women in Burkina Faso observed beneficial effects such as an increase in birth length and an insignificant increase in birth weight of babies born to mothers who consumed fortified food supplement in addition to MMN supplements compared to consuming MMN supplements alone [70]. Yet, fortified food supplements were unable to prevent IUGR (<10th percentile) or LBW (<2500 g) in offspring. Although causation was not established in either study, food-based intervention should also be considered an attractive intervention method, providing another avenue and possibly more accessible methods to improve maternal nutritional status. 

## 5. Omega-3 Fatty Acids 

Omega-3 (*ω*-3/*n*-3) and omega-6 (*ω*-6/*n*-6) fatty acids can be obtained from the diet in their derivative forms, *α*-linolenic acid (ALA) and linoleic acid (LA), respectively. These later become converted by the body into longer chain fatty acids including docosahexaenoic acid (DHA), eicosapentaenoic acid (EPA), and arachidonic acid (AA) [71]. The conversion process is quite slow in humans, and it has since been discovered that EPA and DHA are present in fish oils and AA is present in the phospholipids of grain fed animals [72, 73]. Fatty acids possess a critical structural role in cell membranes and are the parent compound for eicosanoid production [71]. Depending on the parent compound for eicosanoid production, omega-3 and -6 fatty acids play opposing physiological roles. Large amounts of eicosanoids production derived from omega-6-derived LA leads to biologically active metabolic products and upon accumulation can contribute to the formation of thrombus and inflammatory disorders [71], while omega-3 fatty acids derived from fish oils have been shown to exhibit anti-inflammatory effects [71, 74]. One of the first health benefits stemming from its consumption was observed in a study demonstrating an inverse relationship between fish consumption and the risk of coronary heart disease [75]. Subsequent studies treating hyperlipidemic patient populations with high doses of fish oil have also demonstrated the lipid lowering effects of omega-3 fatty acids [76]. The collective and pervasive actions of omega-3 fatty acids may contribute to the prevention of coronary heart disease and hypertension [72].

Interestingly, omega-3 fatty acids have also been shown to be critical in fetal growth and essential for the development of the retina and brain [77]. Furthermore, in a community-based cohort study of healthy women, an association was found between low maternal concentrations of EPA and DHA and high concentrations of AA and a decrease in both fetal growth and birth weight of approximately 50–60 g and increase risk of IUGR [78]. Researchers emphasized the importance of a balanced fatty acid profile in early pregnancy. 

Controlled studies using omega-3 or fish fatty acids oil intervention during pregnancy have found moderately positive benefits. In a study of 533 healthy Danish women, daily consumption of fish oil tablets appeared to increase the length of gestation without impairment of parturition or growth [79]. Daily fish oil consumption from 20 weeks of gestation until delivery also appeared to decrease preterm delivery and extend the gestational period, in a European multicenter study of high-risk pregnancies [80]. Moreover, DHA supplementation during gestation in a large controlled trial significantly decreased the amount of births before 34 weeks of gestation, but increased the number of postterm births [81]. In an Indian population, with normally poor fish intake, an increase risk for LBW was observed in women who did not consume fish in their third trimester of pregnancy [82]. While Rogers et al. found a statistically significant decrease in the odds of IUGR reduction and omega-3 fatty acid consumption, no association was found between consumption and birth weight [83]. Surprisingly, the beneficial effects did not appear in a group of high-risk pregnancy cases as Onwude et al. found that DHA supplementation did not show a significant improvement in gestational length or birth weight [84]. 

Overall, fish oil supplementation during pregnancy appears to have moderate beneficial effects on pregnancy, especially on the length of gestation by two to three days which could potentially be a method for the prevention of preterm birth [85]. However, researchers warn that increasing gestational period may not be desirable if gestation is prolonged beyond term. The 2010 study by Makrides et al. indicated a decrease in preterm birth with omega-3 fatty acids, but a concomitant increase in the occurrence of postterm pregnancies [81]. Postterm pregnancies are also associated themselves with complications such as increased risk of stillbirth [86]. Secondly, studies have suggested that disproportionate high level of fish intake may decrease birth weight [87, 88]. Collectively, these studies indicate that while omega-3 fatty acid supplementation appears to be a favourable intervention, the dosing of intervention must still be validated. 

## 6. Resveratrol 

Resveratrol, a polyphenol, is a protective molecule produced in response to stress by plants [89]. It is found in foods (i.e. grapes and berries), is readily absorbed and can be measured in human plasma [89–91]. It possesses several biological properties including antioxidant activities, vasorelaxant effects, and anticancer functions [92]. More importantly, postnatal resveratrol treatment has shown to prevent symptoms of the metabolic syndrome in hypoxia-induced IUGR from developing in adulthood [93]. 

Given the protective effects of postnatal resveratrol treatment, studies have been aimed at intervening at an earlier time point. Maternal resveratrol supplementation during gestation decreased fetal death by approximately 40% in a severe hypoxemia model in rats [94]. However, the surviving offspring that were growth restricted did not experience a change in fetal weight compared to control. Notably, resveratrol under normoxic conditions led to a decrease in placental weight also suggesting possible placental dysfunction [95]. Resveratrol intervention during pregnancy appears less effective in preventing the development of metabolic syndrome compared to postnatal intervention, but it conveys other protective properties to offspring. 

 Resveratrol has also been considered to help alleviate preeclampsia, and thus decreasing the risk of associated adverse outcomes. *In vitro* studies have demonstrated that resveratrol decreased the amount of soluble fmls-like tyrosine kinase (SFl-T) or vascular endothelial growth receptor-1 release from placental tissues, trophoblasts, and endothelial cells which are known to be elevated in preeclampsia [95]. Levels of SFl-T under a critical threshold are unable to elicit preeclampsia, thus highlighting a novel target of intervention [96]. 

In summary, resveratrol appears to be a safe therapeutic agent as no severe adverse outcomes were observed in human volunteers and demonstrated lack of teratogenicity in pregnant mice [97, 98]. 

## 7. Melatonin

Melatonin, N-acetyl-5-methoxytriptoamine, was first implicated in diurnal patterning and more recently found expressed at high levels in peripheral tissues [99]. Melatonin is considered an important antioxidant, capable of stimulating antioxidative enzymes, scavenging free radicals including superoxides, hydroxyl radicals, and hydrogen peroxide, and possessing repair capabilities [99–104]. Melatonin does not appear to adversely affect prenatal growth or viability in offspring following short-term exposure after pregnancy has been established [99]. With its widespread antioxidant abilities and lack of apparent toxicity, melatonin appears to be an ideal candidate for intervention use in adverse pregnancies. 

 Richter et al. investigated whether the protective effects of melatonin could improve placental antioxidant capacity in rat pregnancies complicated by undernutrition [105]. Melatonin administration during gestation demonstrated restored body weight of offspring at birth and an increase in some antioxidative enzymes, including manganese superoxide dismutase and catalase [105]. In an ovine model, melatonin promoted vasodilation of umbilical blood flow, which may be a mechanism by which fetal growth could be rescued during a complicated pregnancy [106]. In a second ovine model of under nutrition-induced IUGR, short-term exposure to melatonin during gestation was similarly able to increase umbilical cord blood flow [107]. However, melatonin intervention did not rescue fetal weight in nutrient-restricted ewes. The timing of melatonin administration was also investigated in a rodent model of LPS-induced IUGR [108]. In this study, melatonin was administered either post-LPS injection alone or before and post-LPS injection. It was found that posttreatment with melatonin alone led to a decrease in intrauterine fetal death (IUFD) in a dose-dependent manner while administration of both pre- and postinjections almost completely ablated the risk of IUFD and reversed LPS-induced skeletal development retardation. Although the benefits of melatonin were unquestionable, there was still minimal effect observed on recovering fetal weight [109]. 

## 8. Exendin-4

Exendin-4 (Ex-4) is a 39 amino acid peptide and shares 53% homology with glucagon-like peptide 1 (GLP-1) [108]. GLP-1 stimulates insulin secretion and inhibits glucagon secretion and gastric emptying [110]. GLP-1 has also been found to be essential for normalizing fasting glucose levels in diabetic patients [111, 112]. However, GLP-1 agonists themselves are inefficient at maintaining long-term activation, and other analogues have been since investigated have been more resistant to degradation [113, 114]. Ex-4, an analog of GLP-1, has been shown to exert antidiabetic functions such as decreasing plasma glucose concentration, food intake, body weight, and fasting triglyceride levels [114]. Moreover, Ex-4 is able to elicit an approximately ten-fold greater maximal insulinotropic effect compared to GLP-1 [115, 116]. Ex-4 also binds to endogenous GLP-1 receptor (GLP-1R) expressed in *β*-cells and is a more potent agonist compared to GLP-1 [115, 117].

 The astounding effects of Ex-4 were observed following an increase in pancreatic neogenesis and differentiation of *β*-cells in rats after a partial pancreatectomy [118]. Clearly, Ex-4 is an extremely appealing therapeutic agent for the treatment of diabetes. Ex-4 has been used to treat type II diabetes patients that were previously unable to reach normal glycemic control even when using maximal dosages of metformin [119]. Exenatide, a synthetic form of Ex-4, was able to improve glycemic control, proinsulin to insulin ratio, without any of the risks of other antidiabetic drugs [119]. Furthermore, it was shown that exenatide led to a prompt reduction in both fasting and postprandial glucose levels in diabetic patients [120]. The insulinotropic effects and suppression of glucagon observed appeared to be glucose-dependent, negating the risk of hypoglycemia and improving the safety of exenatide use [120]. Interestingly, IUGR rat offspring have been shown to be at risk of developing type II diabetes in adulthood and have a decreased amount of *β*-cells long-term [121]. The use of Ex-4 could theoretically ameliorate glucose regulation and decrease the risk of developing type II diabetes in these offspring. 

 Using rodent models, short-term administration of Ex-4 immediately following birth demonstrated normalization of glucose tolerance and rescue of eventual *β*-cell mass decline [122]. These findings were encouraging, and the mechanisms underlying the prevention of diabetes in an IUGR rat model have been investigated. Ex-4 was able to normalize Pancreatic and duodenal homeobox 1 (Pdx-1) transcription, which is a transcription factor necessary for *β*-cell function and development, while also permanently reversing an aberrant Pdx-1 chromatin environment [123]. It has also been shown that Ex-4 is able to regulate the vascular environment surrounding *β*-cells. The vascular environment plays an important role in normal pancreatic function through its ability to produce signals for differentiation and development, and the delivery of nutrients to *β*-cells. IUGR offspring have decreased islet vascular density, weeks before a loss of *β*-cell mass [124]. Researchers suggested that the vascularity of the pancreas is extremely important in determining the amount of *β*-cells present in offspring. Following short-term neonatal exposure to Ex-4 in IUGR rats, islet vascularity was promptly restored to control levels [124]. It is clear that Ex-4 and exenatide as therapeutic agents should be further explored in the prevention of developing diabetes from an adverse *in utero* environment.

## 9. Nuclear Receptor Agonists

Nuclear receptors represent the largest family of transcription factors found in metazoans, binding to steroid hormones, fat-soluble vitamins, along with oxysterols and bile acids from the diet. Although the roles of many nuclear receptors are well defined in adults, very little is known about their role in fetal development and long-term disease. The use of nuclear receptor agonists as therapeutic intervention in animal models of IUGR is a novel approach that is only just being explored. The peroxisome proliferator-activated receptor gamma (PPAR*γ*), another lipid-sensing nuclear receptor, has been investigated as a target for intervention in neonatal life. PPAR*γ* is a key target of insulin-sensitizing drugs thiazolidinediones and is involved in adipocyte differentiation [125]. PPAR*γ* agonist-treated IUGR female offspring showed insulin-sensitizing effects; however, offspring exhibited severe hypoglycemia as well [126]. 

Our recent studies have demonstrated that in maternal protein restriction (MPR) in rats during pregnancy and lactation, the offspring are low birth weight offspring, with permanent elevation in circulating cholesterol and impaired glucose homeostasis [15, 127]. Moreover, these MPR offspring were characterized by a diminished expression of the nuclear receptor, LXR*α*, and therefore, deregulated expression of LXR*α*-target genes [15, 127]. Given the regulatory role of LXR in cholesterol, triglyceride, and glucose homeostasis [128–131], it is conceivable for the use LXR agonists *in vivo* to improve the expression of LXR target genes and rescue the offspring from undernutrition. In hopes of ameliorating LXR target genes, LXR agonists (GW3695) were administered from postnatal day 5 to 15. Interestingly, by three weeks of age posttreated, LXR agonist-treated offspring had decreased circulating cholesterol : HDL ratios, concomitant with increased LXR*α* and Cyp7a1 expression, and the critical LXR-target enzyme involved in cholesterol catabolism [132]. Furthermore, this was also associated with a more permissive chromatin environment at the promoter region of *Cyp7a1* [132]. These results suggest that maternal protein restriction insults *in utero* are reversible and future studies will focus on the effects of neonatal LXR agonist intervention in adulthood along with the possibility that glucose impairment could be reversed as well. To date, our preliminary data highlights the promising role of nuclear receptors as therapeutic agents in reversing early programming of long-term disease. 

## 10. Conclusion

 As we elucidate the molecular mechanisms underlying the early programming of adult disease, we come closer to not only understanding the development of these diseases but in preventing their onset as well. This paper highlighted some of the current nutritional and pharmacological approaches to date, indicating their short- and long-term beneficial and detrimental effects. Most likely, the most promising targets for the investigation of early programming in the near future will be compounds, which will target common transcription factors (e.g., nuclear receptors) involved in multiple pathways. For example, given that LXR*α* plays a major role in cholesterol, lipid, and glucose homeostasis, it becomes an attractive auspicious candidate for therapeutic targets. In the meantime, known dietary supplements appear promising, even while the dose and frequencies of intervention are still under great investigation. The onus for us remains to further understand the window of opportunity in perinatal life for intervention, which can vary depending on the pregnancy-associated insult. 
